# Sequential management of tibial fractures using a temporary unicortical external fixator

**DOI:** 10.1051/sicotj/2018035

**Published:** 2018-09-07

**Authors:** Anne-Pauline Russo, Alexandre Caubere, Ammar Ghabi, Antoine Grosset, Philippe Mangin, Sylvain Rigal, Laurent Mathieu

**Affiliations:** 1 Department of Orthopedic Surgery, Traumatology and Reconstructive Surgery, Percy Military Hospital, Clamart France; 2 Department of Orthopedic Surgery and Traumatology, Sainte-Anne Military Hospital, Toulon France; 3 Department of Surgery, French Military Health Service Academy, École du Val-de-Grâce, Paris France

**Keywords:** Damage control orthopedics, Open tibial fracture, Unicortical external fixator, Intramedullary nailing

## Abstract

*Introduction*: The development of damage control orthopedics (DCO) procedures has led to the development of temporary unicortical external fixators (TUEFs) intended to limit deep infectious complications and facilitate early conversion to internal fixation.

*Methods*: A retrospective study was conducted in two French military trauma centers, including on patients being treated for tibial fractures with a TUEF (UNYCO^®^ − Orthofix^®^) followed by an early conversion to intramedullary nailing.

*Results*: Eleven patients with an average age of 41 were included between September 2015 and June 2017. A total of 12 TUEFs were implanted for one closed fracture and 11 open fractures, including one type I, eight types II, and two Gustilo types IIIB. The indication of DCO was related to hemodynamic instability in three cases, to the severity of soft tissue lesions in eight cases, and to the context of treatment in one case. The conversion to IM nailing was made after an average of 7.6 days. No significant loss of reduction was observed until internal osteosynthesis, which was performed with “fixator in place” in ten cases. The coverage of Gustilo type III injuries was performed by free flap transfers at the same time as IM nailing. All the patients were reviewed with an average follow-up of 16.5 months. Bone union was achieved in all cases. Two IM nailing dynamizations were carried out, but no bone grafting was required. Two cases of pandiaphysitis were observed and treated without functional complications.

*Discussion*: Despite a limited number of patients, this study demonstrates the reliability of the TUEF to maintain the reduction of tibial fractures and facilitate early conversion to IM nailing. Unicortical fixation does not prevent septic complications related to the severity of soft tissue injuries.

## Introduction

Although the “urgent, complete, and definitive” osteosynthesis of tibial fractures is applicable in most cases, the temporary fixation of tibial fractures must be considered in three different situations: for high-energy open fractures that require iterative procedures; severely traumatized patients with associated vital lesions, with the goal of limiting surgical aggression; in a precarious health context by limiting technical means or massive casualty situations [1,2]. This tactic is part of a strategy of damage control orthopedics (DCO), whose objective is to ensure the fast and temporary initial stabilization of the tibia, allowing for deferred definitive fixation without limiting the technical choices. Whereas a simple plaster orthopedic brace is conceivable in the case of isolated closed fractures, temporary external fixation is necessary for the stabilization of open fractures, segmental fractures, and in polytraumatized patients [2,3]. In general, the definitive fixation of the tibia with intramedullary nailing (IM nailing) is preferred to facilitate reduction, healing, and functional recovery, including in Gustilo type III fractures [4–8]. However, a conversion to definitive, stable, and progressive external fixation is often requested in high-energy open fractures, especially in military practice [1,2,9,10].

The use of a temporary fixator does have its drawbacks, especially if secondary internal osteosynthesis is planned. One of the main limitations is the penetration of the fixator screws into the medullary cavity. The screws bring the medullary cavity into contact with the external environment, presenting a risk of infection during the conversion to IM nailing [11]. To overcome this problem, pinless fixators were developed in the 1990s to provide temporary stabilization of open tibial fractures, but their use has always been marginal [12,13]. Temporary unicortical external fixators (TUEFs) were recently developed in line with this concept [1]. The principle is based on the same specifications as pinless fixators. It is a matter of respecting the intramedullary space by offering high-quality unicortical anchorage using a simple, fast, and reliable implantation technique. Although the main advantage of this fixator is to limit the risk of deep bone infection, it also facilitates the secondary nailing of the tibia that can be carried out with the fixator in place.

The objective of this study was to report the preliminary results of the clinical use of a TUEF for the sequential management of tibial fractures as part of DCO procedures.

## Patients and methods

### Study population

A retrospective study was conducted in two French military trauma centers between September 2015 and June 2017, on patients treated in accordance with DCO principles for open or closed tibial fractures. During this period, tibial TUEF was used for all the patients requiring a DCO procedure. The patients included in the study presented diaphyseal fractures stabilized in emergency with a TUEF (diaphyseal tibia UNYCO^®^- ORTHOFIX^®^), with an early conversion to IM nailing. Patients with tibial fractures extending to one of the epiphyses, and those treated with conventional bicortical external fixator in the initial phase, were excluded.

### Unicortical fixation system

**Figure 1:**
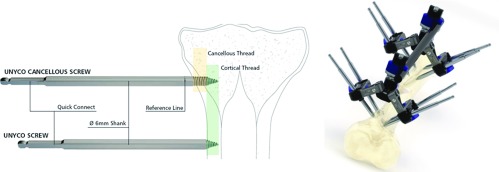
Principles of UNYCO unicortical external fixation^®^.

The TUEF screws used have a specific conical design that allows them to have a deep unicortical implantation without penetrating the tibial medullary cavity. In this respect, they are different from the clips of pinless fixators, which remain on the surface of the bone. The screws are inserted thanks to a motor fitted with a torque limiter that automatically stops before reaching the first cortical bone. Stability is given to each of the two clamps by connecting four converging screws. This multidirectional implantation makes it easier to secure the fixator and limits the risk of damaging any nearby musculotendinous or vasculo-nervous elements (Figure 1).

### Surgical technique

The initial external fixation was always performed under general anesthesia to facilitate the diagnosis of a potential postsurgical compartment syndrome. In the case of open fractures, the procedure began with the meticulous debridement and thorough irrigation of the medullary cavities of the tibia, followed by the setting up of new surgical drapes and changing of gloves and instruments. The two TUEF clamps were positioned independently, proximally and distally of the fracture. The clamps supported four screws each (two anterior screws on the tibial crest and two medial screws) and were connected by a carbon bar measuring 12 mm in diameter. Each unicortical screw had a specific conical design and a 6 mm diameter (Figure 1). Predrilling was not required. The clamps were tightened after the reduction of the fracture. It was possible to add a unicortical intermediate screw to maintain a large third fragment. For open fractures, an amoxicillin/clavulanic acid as antibiotic prophylaxis was prescribed for 72 h, which could be extended until a coverage flap was achieved.

**Figure 2:**
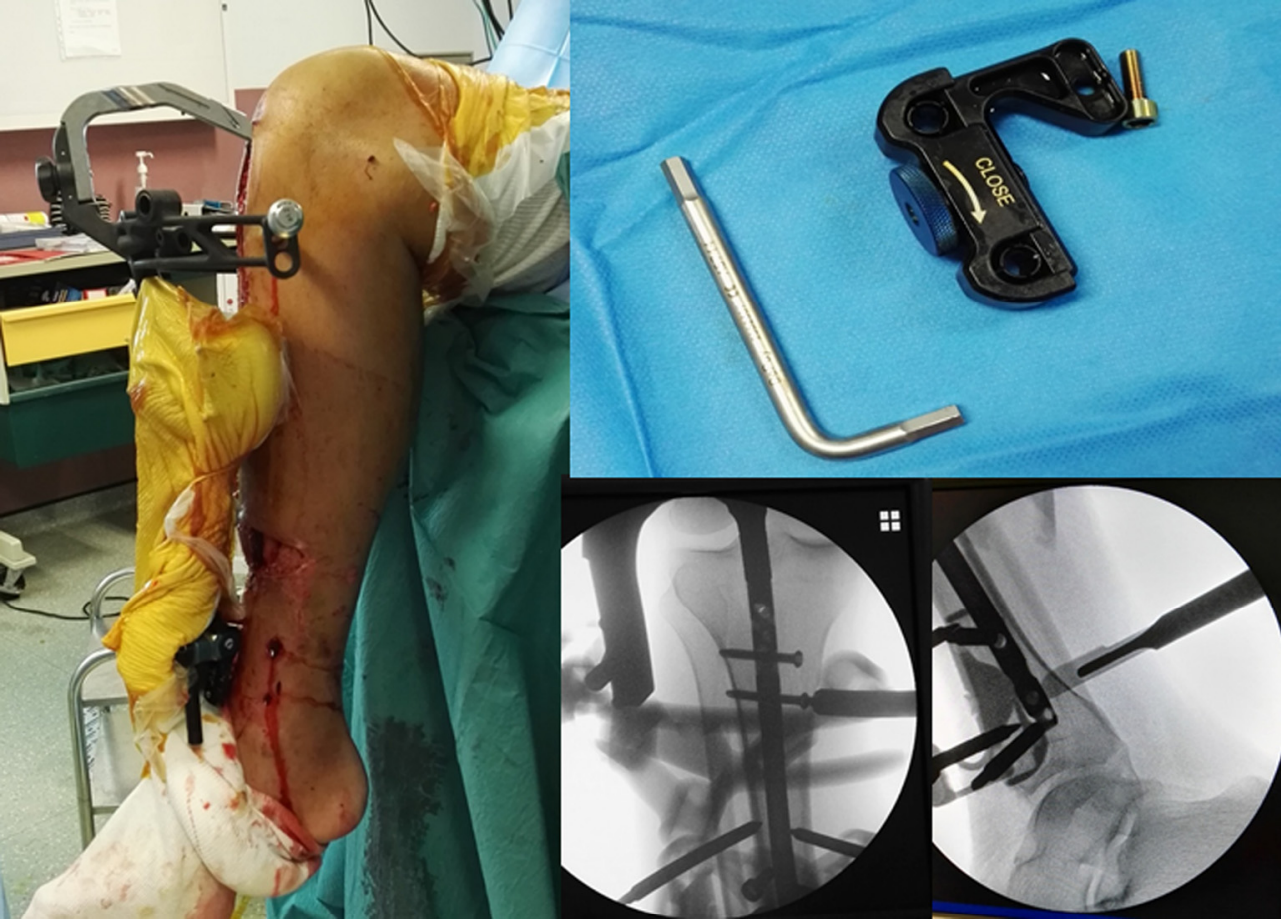
Removing the medial half of the clamps of the fixator makes it easier to lock the nail.

In the absence of signs of infection on the fixator screws or at the fracture site, locked IM nailing with reaming was performed as early as possible. When the initial reduction was optimal, the unicortical fixator was included in the sterile field and kept in place until the end of the procedure. The fixator was disinfected using an iodine-based solution, then wrapped in an adhesive surgical drape. No axial traction was needed. Depending on the location of the clamps, locking the nail may have required the medial half of the clamps to be removed (Figure 2). At the end of IM nailing, the TUEF was removed and the screw holes left for controlled wound healing. In some cases, skin reconstruction with flaps was carried out at the same time, after systematic bacteriological sampling.

### Evaluation methods

The pre-surgical parameters included the age and sex of the patients, the traumatic mechanism, the type of soft tissue injury based on Gustilo, as well as the associated lesions [4]. The period between the two procedures was analyzed, as was the number of iterative debridements before the open fractures were covered. The evaluation of the results concerned the maintenance of the reduction by the TUEF before definitive fixation; its use and effectiveness in maintaining the reduction during the IM nailing procedure; the occurrence of early or late infectious complications; bone union and functional outcome at the last follow-up. An optimal initial reduction was an anatomical reduction of the tibia fracture. A significant loss of reduction between the two surgical steps was defined as a displacement of the tibia fracture superior to 10° on frontal or anteroposterior X-rays. The data were collected using Excel software (Microsoft, Redmond, Washington, USA), which also made it possible to calculate averages and standard gaps.

## Results

### Patient demographics

Eleven patients of average age 41 ± 24 years (range: 18–81 years) were included in the study period. There were nine men and two women. The traumatic mechanism was a road accident in 10 cases, involving seven motorcyclists and three pedestrians hit by a car. The last patient was struck by a train while at work. One patient had a fracture in both legs: 12 fractures were therefore treated in total (Table 1).

**Table 1:** Demographic and treatment parameters.

Patient	Age (years)	Fracture type	Coverage procedure	Time until nailing (days)	Loss of reduction before nailing	Follow-up (months)	Bone union	Complication/revision
1	25	Gustilo II	Direct closure	9	No	21	Yes	Late pandiaphysitis
2	21	R Gustilo II	Direct closure	8	No	29	Yes	Nail dynamization
		L closed	NA	8	No	29	Yes	No
3	49	Gustilo IIIB	ALT flap	7	No	22	Yes	No
4	33	Gustilo II	Direct closure	5	No	13	Yes	No
5	81	Gustilo I	Direct closure	5	No	5	Yes	No
6	18	Gustilo II	Direct closure	7	No	16	Yes	Nail dynamization
7	77	Gustilo IIIB	ALT flap	6	No	18	Yes	Pandiaphysitis
8	29	Gustilo II	Direct closure	7	No	18	Yes	No
9	21	Gustilo II	Direct closure	8	No	9	Yes	No
10	72	Gustilo II	Direct closure	14	No	8	Yes	No
11	27	Gustilo II	Direct closure	7	No	11	Yes	No

### Assessment of the lesion and indication of the DCO

This series consisted of one closed fracture and 11 open fractures, including one type I, eight types II, and two Gustilo types IIIB (Table 1). The tibial fracture was isolated in three cases, but eight patients presented associated lesions: three were polytraumatized and five had multiple fractures without hemodynamic instability. The indication of the DCO procedure was chosen due to the severity of the soft tissue lesions in eight cases, the hemodynamic instability of the patients in three cases, and the technical impossibility of performing IM nailing in one case.

### Perioperative data

For open fractures, debridement was always performed within six hours of the trauma, and the reduction was carried out using forceps before the fixator was implanted. For the closed fracture, the reduction was carried out with external maneuvers based on the principle of osteotaxis by manipulating the clamps of the TUEF. A standard diaphyseal assembly was used in nine cases, intermediate screws were added for two comminuted fractures, and ankle bridging was associated due to an underlying open tibiotalar dislocation. Fasciotomy was not necessary. Direct cutaneous suture was possible for Gustilo type II fractures and negative pressure wound therapy were used for type III. For the latter, iterative debridement was carried out systematically 48–72 h after the trauma.

**Figure 3:**
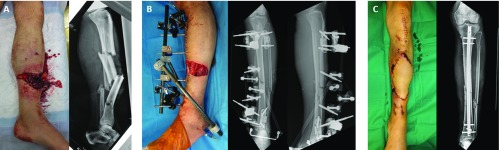
Sequential treatment of a Gustilo IIIB open fracture: initial aspect (A), temporary external fixation and negative wound pressure therapy (B), nailing and free flap coverage at D+6 (C).

External fixation was converted to IM nailing in all cases after an average period of 7.6 ± 2.3 days (range: 5–14 days). An early pulmonary embolism delayed nailing in one case. No significant loss of reduction was noted in the period between temporary and definitive fixation. The TUEF was left in place until the end of the locked IM nailing procedure in ten cases, and there was no perioperative loss of reduction. In two cases, the fixator was removed before the nail was implanted because the reduction of the fracture was not optimal after the first surgical intervention. For both Gustilo type IIIB fractures, coverage with free anterolateral thigh (ALT) flap was performed at the same time as the IM nailing (Figure 3).

### Complications and results at review

Patients were reviewed after an average follow-up period of 16.5 ± 8 months (range: 5–29 months). No complications were noted immediately after the IM nailing procedure, but tibial pandiaphysitis occurred three months after nailing in a 77-year-old diabetic patient (case n ° 7) with a severe Gustilo type IIIB fracture. The diagnosis was made after a pus discharge under the ALT flap. The treatment involved changing the nail combined with prolonged targeted antibiotic therapy. Two other patients were operated again to dynamize the IM nailing due to delayed healing. Lastly, one patient (case n ° 1) presented late pandiaphysitis following the scheduled removal of the nail 16 months after a Gustilo type II fracture. There was no clinical or biological sign of infection before the nail removal. However, three days later the patient suffered an abnormal knee pain with a suspect fluid discharge through the operative wound. Two iterative tibial reamings and prolonged antibiotic therapy were needed to treat the infection.

**Figure 4:**
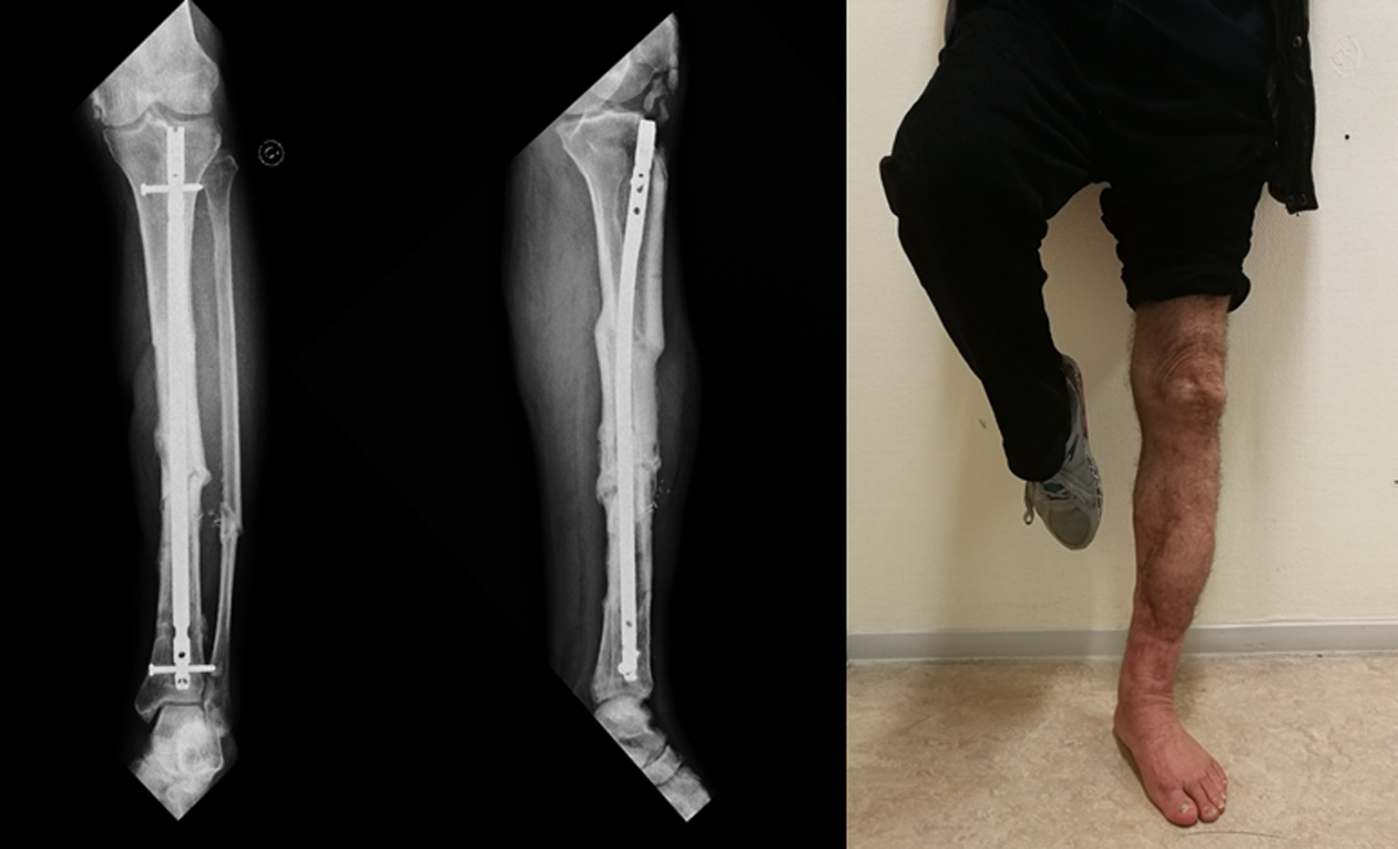
Bone union and functional result at last follow-up despite an early pandiaphysitis (same patient as in Figure 3).

At the last follow-up, bone union was achieved in all cases (Table 1). No significant malunion was observed, but one patient exhibited a shortening of 1 cm linked to the excessive impingement of a comminuted fracture site during IM nailing. No infectious recurrence was observed following treatment for pandiaphysitis (Figure 4). All non-retired patients had returned to their jobs, except for a polytraumatized patient who had neurological complications from a severe head trauma.

## Discussion

DCO is a three-stage surgical tactic that includes: 1 – hemorrhage control, infection prevention, and temporary osteosynthesis of the fractures in emergency; 2 – resuscitation phase or monitoring of pluritissular lesions; 3 – definitive treatment after stabilization of the general condition of the patient and the local state of the limb. The term damage control, which strictly speaking corresponds to urgent lifesaving procedures, is currently used to designate these three stages [1].

Indications of DCO to the tibial segment are well identified and illustrated in this study. They mainly concern open fractures of Gustilo types II and III, given that the development of soft tissue lesions is difficult to predict in emergency cases and requires a secondary reevaluation before the ideal osteosynthesis method is chosen. DCO can also be used when the general condition of the patient or the health context are not suitable for performing IM nailing [1–3]. This is reflected in this series by the use of a temporary EF for closed fractures. When a conventional fixator is used, this last indication may seem excessive compared to simple plaster immobilization. The TUEF is useful here because, while being minimally invasive, it allows for good-quality reduction by osteotaxis (much greater than the one obtained in a cast) and facilitates the detection of a potential compartment syndrome given that the leg remains accessible. These arguments explain the frequent use of temporary external fixation for closed tibial fractures prior to the air evacuation of soldiers deployed in external operations [2]. The only disadvantages of the TUEF in this indication are the additional cost (about 2000 euros for the Galaxy diaphyseal UNYCO© tibia kit with single-use power drill torque limiter) and the presence of scars at the screw implantation sites.

The sequential treatment of Gustilo types II and III has been the subject of numerous studies, especially since the optimum fixation method for these fractures remains debated between external fixation and IM nailing [5–8, 11, 14–17]. While the rate of malunion appears to be lower with IM nailing, not all meta-analyses show differences in terms of infectious complications and nonunion between the two techniques [8,17,18]. IM nailing does have advantages over external fixation: better clinical tolerance, easy access to soft tissue, better control of axes and rotation, and early mobilization and recovery [15,16]. The “fix and flap” strategy is thus used in some centers that perform radical debridement and internal fixation in emergency cases, followed by immediate or very early skin reconstruction (within 72 h) [15,19]. Although this strategy allows for a fast-functional recovery, it does present several pitfalls: the difficulty of a definitive debridement adapted to the initial phase (as soft tissue lesions are progressive in the first days); the need for close collaboration with a team of plastic surgeons; lengthy emergency intervention; and a risk of deep infection in case of deferred coverage [20]. The use of an external fixator followed by an IM nail has thus been proposed to take advantage of the strengths of each technique while limiting their respective disadvantages. In the initial phase, the fixator offers speed of installation and eliminates the need to provide immediate coverage of the fracture site, which instead must be done within seven days [1,15,21]. However, the use of the fixator exposes the medullary canal to contamination, which may lead to pandiaphysitis at the end of the IM nailing procedure. For this reason, it is recommended that the conversion be performed early, i.e. before the 15th post-traumatic day [2,10,15]. Beyond this period, it becomes a late conversion that needs to be carried out in two stages: the fixator is first removed, the screw holes are scraped, an orthopedic brace or a traction is put in place, and the nailing procedure is carried out once the screw implantation sites have healed [1,2].

The use of a fixator whose screws do not penetrate the medullary cavity can limit this problem, but it comes with other disadvantages. Several factors explain the marginal use of pinless fixators: insufficient resistance of the clips and low stability of the assembly (sources of loss of reduction), a risk of cutaneous necrosis around the clips, a delicate placement, and an invasiveness that hinders the creation of the flaps [11,14,22,23]. This study demonstrates that most of these disadvantages have been overcome with the TUEF. First, there was no loss of reduction between the two operating times, nor during the IM nailing procedure. The stability of the assembly therefore seems to be sufficient to maintain the reduction for a period of less than 15 days. It should be noted, however, that all patients remained hospitalized between the two interventions, and that losses of reduction may have been observed if they had been allowed to return home with the fixator. The implantation of the screws was never a problem due to their multidirectional orientation with a displacement of 10°, and no cutaneous necrosis was noted around the screws. In our practice, the fixator did not hinder the creation of flaps where it was removed in advance. This preliminary series confirms that, compared to a conventional fixator, the TUEF has advantages at all three stages of the DCO procedure. In the initial phase, implantation is simple and fast (it takes typically 10–15 min) without requiring an image intensifier to control the depth of screw insertion. In the intermediate phase, it prevents the occurrence of infections in the intramedullary canal while maintaining the reduction effectively. Finally, provided a satisfactory initial reduction is achieved, it can be maintained during the secondary IM nailing to shorten this procedure, which is beneficial when a coverage flap is being created at the same time. It is also possible to perform a minimally invasive plate osteosynthesis by removing the screws implanted on the medial side of the tibia.

The results obtained at the end of the secondary IM nailing in this series are comparable to the data in the literature and confirm its effectiveness in limiting the occurrence of malunion and facilitating functional recovery [18,24]. Excluding the need to change a nail due to an infection, all fractures (open or closed) healed without bone supply. It was, however, necessary to dynamize the IM nailing in two cases. The two cases of pandiaphysitis cannot be attributed to the use of the TUEF. The case that occurred after the type IIIB fracture is most likely linked to the severity of the soft tissue lesions and possibly to a debridement fault. The indication of conservative treatment was also questionable in this elderly, diabetic patient. The origin of the late infection after the scheduled removal of the material is more uncertain: it may be a matter of contamination during extraction of the material or an undetected underlying chronic infection. Carrying out IM nailing by keeping a fixator implanted for several days is one of the questionable points of this tactic. Indeed, despite careful disinfection and isolation of the body of the fixator as the surgical drapes are being set up, asepsis cannot be completely guaranteed during this type of procedure, especially if parts of the clamps need to be removed to lock the nail. Finally, the use of IM nailing with reaming is open to criticism when it comes to open fractures. Many authors recommend the use of unreamed nails in this indication to limit the risk of infection and bone necrosis [25]. However, this point remains controversial because reamed nails provide better stability [7]. The meta-analysis by Papakostidis et al. [26] demonstrated that, for Gustilo type III fractures, the healing rates were higher with reamed IM nailing and that there was no difference in terms of infection between reamed and unreamed nails.

This preliminary study has obvious limitations related to the small size of the series, which makes it impossible to draw conclusions on the reliability of this treatment strategy. The effectiveness of the TUEF in maintaining the reduction between the two DCO operating times remains to be confirmed. To this end, a multicenter prospective study was launched to evaluate the biomechanical behavior of the screws over time and to determine the limits of use of this fixator.

To conclude, the temporary external unicortical fixation of the tibia seems to be a preferred technique in the context of a DCO procedure. For closed fractures, it allows for high-quality reduction by osteotaxis while making it possible to monitor the fascial compartments. For type III open fractures, it allows the “fix and flap” tactic to be deferred for a few days to carry out the most suitable treatment in optimal conditions. Further investigations are required to confirm the biomechanical reliability of this anchorage in clinical practice.

## Conflict of interest

The authors declare personal connections with Orthofix^®^.

## Disclaimer

The views expressed in this manuscript are those of the authors and do not reflect the official policy or position of the French Medical Health Service.
